# The Efficacy of Subchondroplasty for the Treatment of Knee Pain Associated with Bone Marrow Lesions

**DOI:** 10.51894/001c.11767

**Published:** 2020-01-30

**Authors:** Nathan M Krebs, James L. Kehoe, Michael J. Van Wagner, Carlos Rios-Bedoya

**Affiliations:** 1 Cincinnati Sports Medicine and Orthopaedic Center Mercy Health; 2 McLaren Macomb Medical Center; 3 Division of Scholarly Inquiry McLaren Health

**Keywords:** arthroscopy, subchondroplasty, osteoarthritis, bone marrow lesion

## Abstract

**CONTEXT:**

Symptomatic bone marrow lesions on MRI in patients with knee osteoarthritis are strongly associated with progressive deterioration of the joint and an increased risk of progression requiring joint replacement surgery. This study evaluates the efficacy of knee arthroscopy with adjunctive subchondroplasty (i.e. cartilage stabilization) to improve self-rated visual analog scale (VAS) pain scores, rate of conversion to arthroplasty, and patient satisfaction levels.

**METHODS:**

A retrospective chart review and phone survey was performed on 12 patients who had undergone knee arthroscopy with adjunctive subchondroplasty for knee pain associated with chronic subchondral bone marrow lesions on MRI. Follow-up for the 12 patients was 36 months on average (range of 12 to 51 months), self-reported paired preoperative and postoperative VAS scores were analyzed in addition to rate of conversion to arthroplasty and patient satisfaction.

**RESULTS:**

The results demonstrated statistically significant reductions in mean preoperative VAS scores versus six-week postoperative VAS scores from 7.58 to 1.83 (p < 0.001) in addition to significant reductions in mean preoperative VAS scores to final postoperative VAS scores from 7.58 to 1.60 (p < 0.001). There was no statistically significant association (p > 0.05) with patients’ demographic and clinical data (e.g., age, height, weight, BMI, length of symptoms) and rate of revision to total arthroplasty after receiving the arthroscopic subchondroplasty procedure. Out of the 12 patients, two (16.7%) patients went on to conversion to total knee arthroplasty.

**CONCLUSIONS:**

In this series, knee arthroscopy with adjunctive subchondroplasty for the treatment of osteoarthritis with symptomatic bone marrow lesions was associated with clinically significant improvements in VAS pain scores. Furthermore, patients who underwent subchondroplasty had a low rate (16.7%) of conversion to total knee arthroplasty at 36-month follow-up.

## INTRODUCTION

Osteoarthritis, also known as degenerative joint disease, is the most common disease affecting the knee and leads to approximately 700,000 total knee replacements (i.e., arthroplasty) performed annually in the United States.^1^ Arthroplasty involves removing the diseased articular portions of the knee and replacing them with a prosthetic joint consisting of metal and plastic.

Atraumatic subchondral bone marrow lesions (BML) of the knee identified on magnetic resonance imaging (MRI) have been shown to be associated with progressive deterioration of articular cartilage and severe arthritic changes to the joint.^2–6^ A BML is an area of increased signal intensity in bone as seen on T2 sequences on MRI. These findings have been termed “insufficiency fractures” and are thought to play a role in the development of pain associated with osteoarthritis, as the subchondral bone is richly innervated with nerve endings.^7–10^

In 2008, Scher and associates found that in patients who have knee osteoarthritis, there was a nine-fold increase in the rate of progression to total knee arthroplasty within three years when there were symptomatic subchondral BML identified on MRI compared to osteoarthritis patients without an associated BML.^11^ Total knee arthroplasty is a major surgery and although the procedure is generally successful, with patient satisfaction rates of up to 88%, arthroplasty can also be associated with significant morbidity.^12^

It has been concluded that knee arthroscopy alone for the treatment of symptomatic osteoarthritis of the knee is not beneficial.^13–17^ When more conservative measures fail, surgical treatment of osteoarthritis often results in arthroplasty.^18^ It has also been hypothesized that stabilizing symptomatic subchondral BMLs with synthetic calcium phosphate bone substitute particles may provide support to the overlying articular cartilage while the subchondral bone remodels.^19–22^ This added stability may help to alleviate the pain associated with these lesions.^23,24^

This procedure is known as a subchondroplasty and is commonly performed in conjunction with knee arthroscopy.^22^ Subchondroplasty remains a relatively novel treatment for osteoarthritis of the knee with associated symptomatic BML, with limited but encouraging data to date supporting its use.^22,25–27^

**Purpose of Study** The primary purpose of this study was to measure the efficacy of the arthroscopic subchondroplasty procedure on three outcomes: a) changes in postoperative visual analog scale (VAS) pain scores (0-10) , b) rate of conversion to arthroplasty, and c) overall patient satisfaction ratings. The secondary aim of the study was to test for possible differences in outcomes of patients with varied demographic and condition-relevant clinical characteristics (age, sex, height, weight, body mass index (BMI), length of symptoms).

## METHODS

A chart review and phone survey were performed on all patients who had undergone a subchondroplasty procedure between January 2014, (i.e., when subchondroplasties were first performed at our institution), and December 2017 by one of the three different orthopedic surgeons at a teaching community hospital. This time frame was selected to study patients that have had at least a 12-month follow-up. The surgeons in this study included Dr James Kehoe, who is the senior author in this study, in addition to Dr Shariff Bishai and Dr Jeffrey Carroll.

After obtaining Institutional Review Board (IRB) approval, eligible patients were contacted by phone and asked to consent to participate in the study. Of the 31 patients who had received a subchondroplasty during the selected study period, only 12 (38.7%) patients were successfully contacted by telephone to participate in the study. There were 2 additional patients that were able to be contacted, but did not wish to participate in the study.

A follow-up phone survey at six weeks after surgery and at the end of the clinical follow-up were conducted to examine patients’: 1) current VAS pain ratings (0-10), 2) history of conversion to total arthroplasty of the affected knee, and 3) to determine overall patient satisfaction with the subchondroplasty procedure. Patient satisfaction was assessed by asking the following two questions: 1) “Were you satisfied with the results of your subchondroplasty procedure?” and 2) “If you could go back in time, would you still choose to have the procedure?” An additional chart review was performed to collect preoperative and postoperative VAS pain scores and data concerning conversion rates to arthroplasty.

The inclusion criteria included moderate to severe pain for greater than three months; failure of symptom relief with over the counter non-steroidal anti-inflammation (NSAID) medications, corticosteroid injections, hyaluronic acid injections, or physical therapy; the presence of at least one BML in a weightbearing portion of the knee on MRI; patient reported pain within the knee compartment corresponding to BML location on MRI; with pain in the corresponding compartment of greater than 4 out of 10 based on the VAS score.

Patients were excluded from the study if their primary cause of pain was likely due to another pathology as determined by clinical exam or intraoperative findings. Exclusionary criteria included: a) the presence of inflammatory arthritis, b) Kellgren-Lawrence grade four changes consisting of severe joint space narrowing and bone spur formation of the involved compartment, c) gross knee instability, or d) greater than seven degrees of varus (i.e., bow-legged) or valgus (i.e., knock-kneed) alignment. Patients who were found to have significant intra-articular pathology during arthroscopy that could have contributed to their pain were also excluded. No patients that were contacted and wished to participate in this study required exclusion based on the above criteria.

### Surgical Technique

The subchondroplasty procedure was performed in a standard arthroscopic fashion by the three surgeons as previously described in similar studies.^2,25,28–30^ Preoperative MRIs were used to determine the size and location of each patient’s subchondral BML. Knee arthroscopy was first performed to examine the articular cartilage and evaluate the other structures of the knee. A cannula, or thin metal tube, was used with the assistance of intraoperative fluoroscopy to inject (Zimmer brand) calcium phosphate bone substitute particles into the affected subchondral region after using a drill to decompress the lesion. Bone marrow lesions on MRI that correlated with clinical symptoms were treated with 2 to 3cc of bone substitute. No intraoperative complications were reported for any sample patients.

### Postoperative Protocol

Patients were permitted to be full weight-bearing immediately after surgery and were provided standard postoperative instructions for knee arthroscopy. All patients were seen postoperatively at the standard follow-up intervals with release to full unrestricted activities at six weeks. The standard postoperative follow-up included evaluations at two weeks, six weeks, three months, and six months. Patients are then seen one-year postoperatively and annually thereafter. None of the sample patients required formal postoperative physical therapy.

### Data Collection

For all patients, baseline preoperative VAS pain scores were obtained in addition to VAS scores at six weeks follow-up. Dr Krebs and Dr Kehoe recorded documentation for all failed nonoperative treatments in addition to retrospective review of patient anthropometric data, operative reports and imaging. Patients who underwent conversion to total knee arthroplasty did not have their final postoperative VAS scores included in the data analysis. The average final postoperative VAS scores were obtained at 36 months (range 12-51 months).

### Statistical Analysis

Before proceeding with any statistical analysis, the fourth author CRB assessed data outliers, out of range values, and the need for data cleaning and editing by performing a series of frequencies, proportions, descriptive statistics (e.g., mean, median, and standard deviation) and figures (e.g., histograms and box and whisker plots). After this process, any needed data editing and cleaning was conducted. Bivariate analyses were performed to determine any associations between the study explanatory variables (i.e., age, sex, BMI, height, weight, and length of symptoms) and preoperative and postoperative VAS scores.

Such analyses included Fisher exact tests and independent as well as paired Student’s t-tests for categorical and continuous variables, respectively. Fourth author CRB conducted all analyses using Stata/MP 15 statistical software package (Stata Corporation, College Station, TX). The usual 0.05 Type I error threshold for statistical significance was used for all analyses.

## RESULTS

### Patient Characteristics

The average age of patients included in this series was 58 years (SD±17.6) years old (range 26 to 85 years old). The 12 patients included in this study consisted of five males and seven females with a total of three right knees and nine left knees. The average height was 67 (SD±4.3) inches (range 59 to 72 inches) and average weight was 177 (SD±25.0) pounds (range 150 to 228 pounds). The average body mass index (BMI) was 30.3 (SD±7.3) (range 22.8 to 39.5). The average length of symptoms (LOS) before surgery was 7.4 months (SD±2.6) (range four to 12 months). A review of patient characteristics is presented in Table 1.

**Table 1.** Selected characteristics of patients undergoing knee arthroscopy with adjunctive subchondroplasty with and without conversion to arthroplasty

	Total (n=12)	Conversion (-) (n=10)	Conversion (+) (n=2)	p-value
Age (mean±SD)	58.2 (17.6)	58.3 (17.1)	57.5 (27.6)	0.957
Sex (%) Male	58.3	60.0	50.0	> 0.999
BMI (kg/m^2^) (mean±SD)	30.3 (7.3)	31.1 (7.7)	26.3 (4.2)	0.420
Height-inches (mean±SD)	66.6 (4.3)	66.6 (4.6)	66.5 (3.5)	0.978
Weight-lbs (mean±SD)	177.1 (25.0)	179.7 (26.6)	164 (8.5)	0.443

### Patient reported satisfaction and VAS scores

Significant reductions in six-week postoperative VAS scores (1.83) were observed when compared to preoperative VAS scores (7.58) (p < 0.001). Similarly, final postoperative VAS scores (1.92) were significantly lower than preoperative scores (7.58) (p < 0.001). There was no statistically significant difference (p > 0.05) in VAS pain scores between 6-week postoperative (1.83) and final postoperative scores (1.60). Please refer to Table 2 for a summary of the results. The improved postoperative VAS scores after the subchondroplasty procedure were consistent with the overall patient reported satisfaction rate of 92% (11 of 12 patients).

**Table 2.** Paired comparison of VAS score from baseline to six week postoperative and final postoperative visits

Outcome	Baseline VAS (n=12)	Six-week VAS (n=12)	Final VAS (n=10)	p-value
VAS score (mean±SD)	7.6 (1.2)	1.8 (1.7)	--	< 0.001
VAS Score (mean±SD)	7.6 (1.2)	--	1.6 (1.9)	< 0.001

*VAS scores of patients who had undergone conversion to arthroplasty excluded from Final VAS analysis

### Conversion to Arthroplasty

Of the 12 patients included in this series, two (16.6%) went on to an arthroplasty procedure on the affected knee within the time frame studied. Of these two patients, the average length of time from subchondroplasty to conversion to total knee arthroplasty was 9.5 months (range seven to 12 months). There was no association with patient demographic or clinical characteristics (age, height, weight, BMI, length of symptoms) and rate of conversion to arthroplasty after having the subchondroplasty procedure.

The mean postoperative VAS scores at six weeks for patients who underwent conversion to arthroplasty (4.5) was significantly higher than the patients who did not undergo an arthroplasty procedure (1.3; p = 0.009). There was no statistically significant correlation with preoperative or final VAS pain scores and rate of revision. Fishers exact test was performed and did not identify any significant associations with gender, side of knee, surgeon, or satisfaction score and revision to arthroplasty.

## DISCUSSION

The findings from this study demonstrated significant reductions in VAS pain scores at an average of 36-month follow-up in this sample of patients receiving the arthroscopic subchondroplasty procedure. These findings constitute clinically significant changes, as there were over five-point decreases in VAS scores following surgery. Improvements in VAS pain scores have been shown to be clinically significant with changes of at least two points.^31^ Furthermore, only two out of the 12 patients in this series were converted to total knee arthroplasty during the time frame studied.

The current literature has demonstrated favorable results for most patients who receive the subchondroplasty procedure such as improved VAS pain scores and low conversion rate to total arthroplasty within two-year follow-up.^22,25,29,30,32–34^ Cohen et. al.^25^ showed similar findings to our study with statistically significant reductions in postoperative VAS scores and approximately 30% conversion rate to total knee arthroplasty at 24-month follow-up. In 2013, Davis et al^35^ reported preliminary data from a sample of 50 patients at an average of 14.6 months follow-up demonstrating an average of 4.7-point improvement in VAS pain scores with only four patients progressing to total knee arthroplasty. Additionally, patient satisfaction in this same study was generally high with 78% of patients stating they would undergo the procedure again.

Similarly, Bonadio et. al.^28^ demonstrated in a case series of five patients that those who received subchondroplasty showed a significant improvement in functional capacity and improved pain scores from the first week post-procedure to six months postoperatively. In 2013, Farr and Cohen^29^ also found favorable results in their study of 59 patients who underwent the subchondroplasty procedure, demonstrating significant improvement in pain in 75% of the sample. Additionally, the subchondroplasty procedure has been presented as a viable technique in the treatment of chronic osteochondritis dissecans of the knee in stable lesions with persistent pain after extensive non-operative management.^36^

The 2015 Chatterjee et. al.^36^ article challenged the efficacy of subchondroplasty in their series of 22 patients. Based on their results, they advise against the use of knee subchondroplasty for symptomatic BMLs. Their study evaluated the efficacy of the procedure using the Knee Injury and Arthritis Outcome Score (KOOS) and the Tegner Lysholm Knee Scoring Scale, which did find statistically significant improvement in postoperative scores, but did report poor clinical outcomes in 32% of patients at a median of 12 months follow-up. This conclusion has been regarded as controversial as many argue that the Tegner Lysholm Knee Scoring Scale is not an appropriate outcome measure as it has been historically used as a measure after anterior cruciate ligament reconstructions.^25^

For the treatment of knee osteoarthritis with associated symptomatic BMLs, knee arthroscopy with adjunctive subchondroplasty appears to be an effective and less invasive treatment option with minimal risk of complications. In the literature review by Astur et. al.^32^ few surgical complications were found with percutaneous calcium phosphate injection. The most frequent complaint was disproportionate knee pain after surgery, which resolved within 72 hours in all cases. Further reported complications include extravasation of the bone substitute into the joint or soft tissues and deep vein thrombosis in the affected extremity.^25,32^ In our series, there were no reported complications related to the subchondroplasty procedure.

The question has arisen regarding possible technical difficulty in performing knee arthroplasty on patients who previously underwent subchondroplasty. A study by Yoo et al.^37^ found no increased procedural difficulty or any adverse effects in patients who had undergone previous subchondroplasty. Furthermore, there was no increase in the rate of complications in the study group of 22 patients versus the control group. The average follow-up was 23.5 months, leading the authors to state that longer follow-up is needed to truly assess implant longevity.^37^

### Study Limitations

Our work has several limitations that must be considered in the evaluation of this study. First, it is a case series with only 12 patients, which leads to the possibility of selection bias and lack of generalizability. The relatively low number of patients is secondary to the subchondroplasty surgery being a newer type of procedure for treatment of a small subset of patients with BMLs on MRI. Second, there is no control group to compare to the intervention group.

Third, there was a lack of standardization in the collection of postoperative data due to patients missing appointments and not filling out VAS pain scores at regular progressive intervals from the time of surgery. Fourth, there was a general lack of postoperative MR imaging to evaluate the status of the BML following subchondroplasty. Despite these limitations, this study offers the longest follow-up data at 36 months on a set of patients undergoing the subchondroplasty procedure.

## CONCLUSIONS

In this series, knee arthroscopy with adjunctive subchondroplasty was associated with clinically significant improvements in VAS pain scores and a low rate of conversion to total knee arthroplasty in a sample of patients with symptomatic knee BMLs at a minimum of 36-month follow-up.

Future studies are needed to examine the possible improvement of bone marrow edema or signs of subchondral BML remodeling with clinical outcomes. Larger randomized-controlled trials are also needed to truly define the indications and prognostic indicators when considering the use of subchondroplasty for the treatment of symptomatic BMLs.

### Conflict of Interest

The authors declare no conflict of interest.
